# Mr. Low, on Metallic Casts of the Ear

**Published:** 1803-10-01

**Authors:** Robert Low

**Affiliations:** Tavistock-row, Covent-garden


Gentlemen,

THE anonymous attack upon the small communication of mine, which you honoured with an insertion in the fifty-second number of your Journal, although it is very derogatory to my wish to intrude useless controversy upon the public, I look upon myself, in justice to my own feelings, warranted in making a short reply to.—At the time I offered the result of my experiments on taking metallic casts of the ear to public notice, I was very well aware, and I believe observed, that some persons had been successful in performing operations of a similar nature; one of whom, to my knowledge, was so fearful of spreading his information abroad, that, when solicited to communicate the process which had rendered his own experiments fertile, he refused to do it. As I never studied anatomy in Windmill-street, or heard Mr. Cruikshank or Dr. Bailey deliver their lectures on the art of making anatomical preparations, I would by no means presume to say that those gentlemen were not acquainted with a method similar to that which I recommended, and which, as I have before asserted, occurred to me, after making a variety of experiments; I can only observe, that of several gentlemen of great observation, with whom I am intimate, who regularly attended the lectures of the above justly celebrated professors, (one I believe for five years) not one knew any more of injecting the temporal bone, except, that it was requisite it should be calcined in a fire, and have a melted metal poured into its cavities, the nature and composition of which they were totally unacquainted with. To exculpate myself from any imputation of plagiarism, I have only to observe, that nothing in print ever met my eye, nor did any observation in lecture ever meet my ear, inculcating any way similar to what I have recommended: indeed, a very celebrated anatomical lecturer, with whom I was house pupil, was very anxious to become acquainted with a successful method of injecting this organ. These circumstances, with the detestation in which I have held a secret-monger in science, were my sole inducements for laying my plan before the numerous circle of your readers. I have several times used pipe clay as a luting; but from its liability to become detached from the surface of the bone and fly into pieces, as a gentleman, who assisted me in some of my experiments witnessed, I have never been able to succeed with it. As the Paris plaster luting, with the greatest care and attention in previously drying the bone, will sometimes crack, I conceive the crucible enclosure would afford a valuable protection to it. The oxydation of the metal, which always occurs from its exposure to the atmosphere, may be prevented by laquering the cast over with a varnish composed composed of alchohol and gum mastic, or sandarac, in the way that philosophical instrument-makers preserve the metallic surfaces of their instruments.

*Tavistock-row, Covent-garden,* I am, &c.

*August* 3, 1803. ROBERT LOW.

---

### *To the Editors of the Medical and Phyfical Journal.*

Gentlemen,

